# Anisotropic distortion in the perceived orientation of stimuli on the arm

**DOI:** 10.1038/s41598-021-93959-2

**Published:** 2021-07-16

**Authors:** Scinob Kuroki

**Affiliations:** NTT Communication Science Laboratories, NTT Corporation, Kanagawa, Japan

**Keywords:** Sensory processing, Somatosensory system

## Abstract

Mechanoreceptors on the skin are heterogeneously distributed, and the sampling of neural signals in the brain can vary depending on the part of the body. Therefore, it can be challenging for the brain to consistently represent stimuli applied to different body sites. Here, we report an example of a regional perceptual distortion of the tactile space. We used a piezoelectric braille display to examine shape perception on the volar surface of the arm and to compare it to that on the palm. We found that the orientation of perceived stimuli on the arm was distorted in certain areas. In particular, an inwardly-inclined line shape was perceived as being more inwardly-inclined than it actually was. On the other hand, an outwardly-inclined line was perceived accurately. When the same stimuli were applied to the palm, this anisotropic bias was not observed. We also found that changing the posture of the arm changed the angle at which this anisotropic distortion occurred, suggesting the influence of the skin frame of reference on this illusion. This study showed a clear example of how the representation of even simple stimuli is complexly distinct when the stimuli are applied to different body sites.

## Introduction

When a haptic stimulus is applied to the surface of the body, we can represent its spatial properties, such as position and shape. This fundamental function is known to be imperfect: the representational space of touch is sometimes distorted and can differ depending on where the stimulus is applied to the body. The perceived distance between two contact points on the hand or arm is contracted along the proximal–distal axis compared to that between two contact points separated by the same distance along the radial-ulnar axis^1–8^. The perceived location of the stimulus is shifted toward anchor points: a stimulus applied to the back of the hand is perceived as close to the knuckles^9^, and a stimulus applied to the arm is misperceived as being close to the wrist or elbow^2,10^. Stimulus localization was more accurate in the radial-ulnar axis than the proximal–distal axis^10,11^. These distortions must be related to variations in receptor distribution and receptive field size^12,13^; still, not all the findings can be explained by a simple stretching of tactile space from the receptor distribution. For example, the perceived distance between two contact points can be changed by how the contact is applied. When two points are touched at the same time, the distance between them is perceived to be shorter than when they are touched sequentially^9,14,15^. The mechanism of haptic spatial perception and its variations depending on where the stimulus is applied remain elusive.

Haptic spatial perception, especially orientation sensitivity, has been extensively studied in the context of haptic parallelism (e.g.,^22–30^). In the typical setup of previous studies, blindfolded participants were asked to manually turn one physical bar (e.g., an aluminum rod) with their hand so that the bar was set to be parallel to a different reference bar whose angle was fixed. One of the main findings was a haptic oblique effect, originally known as a visual phenomenon^31^, where deviations of the reported/matched angle for the oblique reference bar were substantially bigger than that for the vertical and horizontal reference bars. Furthermore, two bars that were touched by the right and left hands respectively and set to be parallel could deviate significantly from being physically parallel^22,27,29,30^. Although the procedure of these studies included an element of action (of rotating the bar) and not purely sensory processing, their results suggest that the orientation perception of even simple stimuli is distinct when the stimuli are applied to a different body part.

In this study, we will focus on orientation perception on the arm, especially as it differs from that on the hand. As a substitute for hands, arms—especially forearms—have begun to attract attention as an alternative location for information presentation^2,16–21^. The arm has a relatively large and flat surface area that is seemingly suitable for presenting information using spatiotemporal stimuli while preserving free use of the hands. Meanwhile, most previous studies explored the possibilities of haptic information presentation by applying stimuli to fingers and hands. How generalized these findings can be in regard to other peripheral body parts (e.g., arms) remains a question. The number and distribution of receptors differ widely between the hand and the arm, as does the way they are used in daily life. If the brain were to perform the same information processing on the incoming signals to the arm as it did for the hand, this might be inefficient in terms of both computational time and cost. In this study, we address the following two points: (i) whether the orientation of a simple line stimulus that can easily be recognized by the hand can be distinguished by the arm and, if not, (ii) how the arm differs from the hand in this respect. Needless to say, we rarely use our arm to judge the shape or angle of objects. However, since the arm is connected to the hand, which is frequently used to judge the shape and angle of objects, we could possibly extract orientation information through input on the arm. Note that since there are few previous studies that have investigated orientation perception on the arm, this study was conducted in an exploratory manner without setting any strict hypotheses.

In our experiments, we tried to systematically investigate the orientation representation of simple line stimuli applied to the arm using a braille stimulator. We measured orientation perception on the palm as our baseline condition. We compared the results to see if orientation of the stimuli were perceived differently between the two body sites. In addition, we tested four different conditions to ascertain whether stimulus dynamics influence orientation perception. One condition was a static condition in which the line shape was presented all at once and the others were dynamic conditions in which the line was spatiotemporally presented by a moving dot. The participants should perform better in the static condition if they could estimate the orientation more accurately when the stimulus extended over a larger area and/or applied at once. On the other hand, the participants should perform better in the move condition if they could detect the stimulus location more accurately when the stimulus was more locally applied and the detected spatial locations could be temporally pooled and perceived as a shape. To test how the stimulus motion dynamics influence orientation perception, we tested three dynamic conditions. One condition was the shuffle condition where the dot appeared randomly on the bar shape and the other two were move conditions in which the bar shape was presented by the dot moving in one direction (approaching or moving away from the body). The participants should perform better in the move conditions if they predicted the trajectory of the moving dots to estimate the orientation. On the other hand, the participants should perform better in the shuffle condition, in which the dots appear at distant locations, if they could pool the spatial location of the detected stimuli within a short period of time.

## Results

### Experiment 1

**Figure 1:**
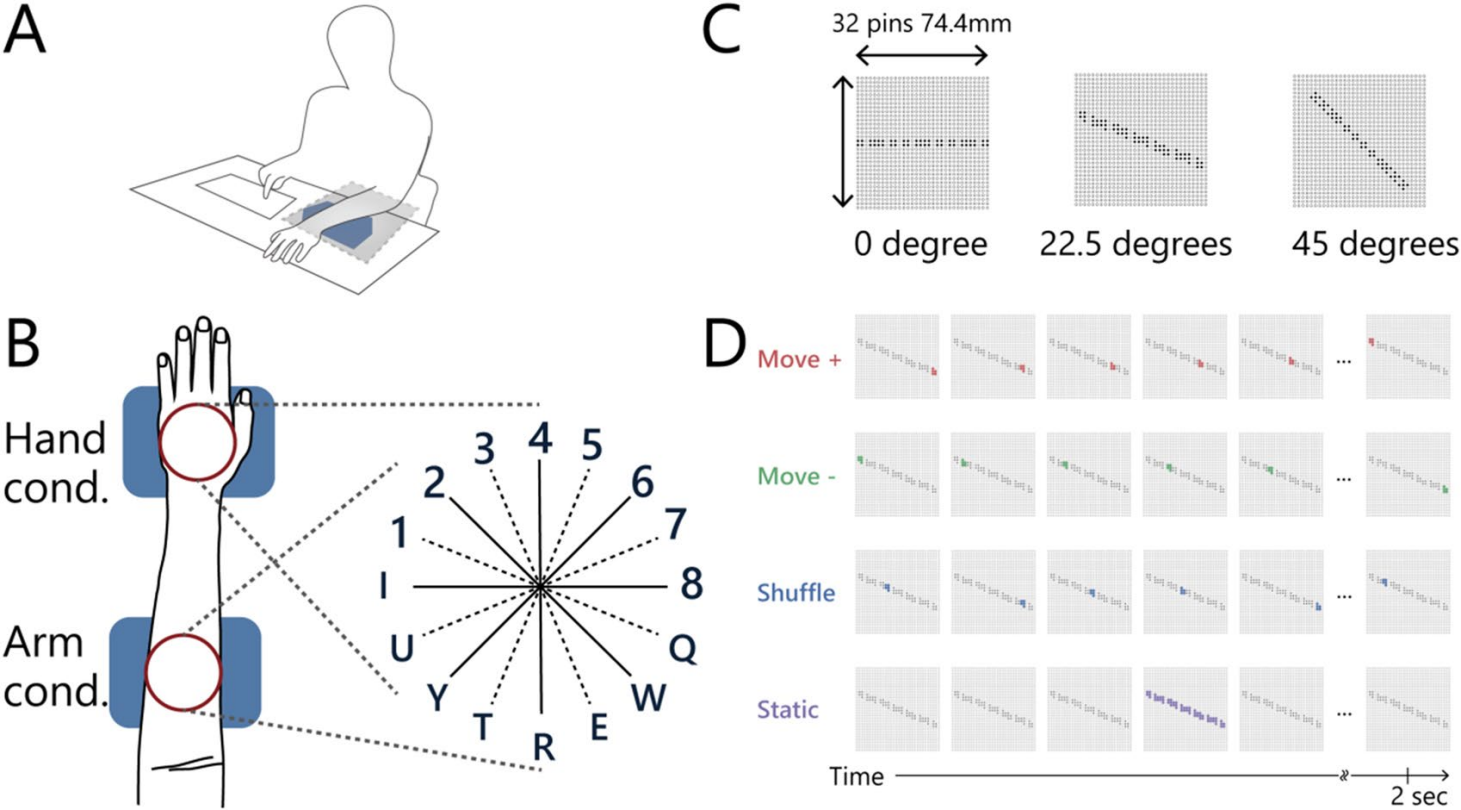
**(A)** View of the setup used in the experiments. The blue box represents the braille stimulator. The gray shaded area represents an occluder placed between the participants’ view and the stimulator. **(B)** The participants reported the perceived angle (both ends) of the stimulus shape with respect to the stimulator surface by pressing two keys. The wheel illustrated on the right was displayed as a response map in front of the participants during the experiments. **(C)** Trajectory of the stimuli. Open circles represent pins that remained in the “off” position for the duration of the stimulus presentation (2 s), and closed circles represent pins that took the “on” position for 0.1 to 0.2 s at some point, depending on the stimulus dynamics. Due to the specifications of the braille stimulator, the pins were not in an exactly straight line under some angle conditions. **(D)** Time course of a 22.5° angle of stimulus under four stimulus dynamics. Black circles represent the stimulus trajectory, and coloured circles represent pins in the “on” position at that time.

The purpose of Experiment 1 was to see the difference in orientation perception when a simple line shape was applied to the palm of the hand and when the same shape was applied to the volar surface of the arm. The experiment was carried out by positioning the arm on a desk with the palm down (Fig. 1A). This position is natural and is similar to the posture normally used when typing.

Participants were asked directly to indicate the angle of stimuli with respect to the stimulator’s surface (Fig. 1B). The stimuli were dots aligned in one of eight possible angles with four different stimulus dynamics: dots appear one by one in one direction (“move + ”), in the opposite direction (“move-”), in random order (“shuffle”), or all at once (“static”) (Fig. 1C,D). The stimulus duration in the static condition was instantaneous since all the dots were presented simultaneously, while that in the dynamic conditions was longer since the dots were presented one by one. Note that the end-to-end distance of all the dots was longer than the two-point discrimination threshold for the forearm^21^. For each stimulus dynamic and each angle of the presented stimuli, the response was calculated for each participant and then averaged across participants.

**Figure 2:**
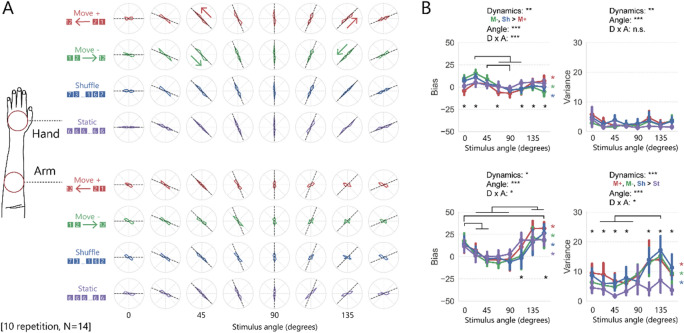
Results of Experiment 1. **(A)** The average rate of the stimulus orientation reported by the participants. Each column represents the results obtained with a stimulus presented at 0° to 157.5°. A stimulus at 0° was defined with respect to the front edge of the braille stimulator placed parallel to the table. Black dashed lines represent the angle of the presented stimuli, and each colored line represents the proportion of the reported angle under each condition. The red and green arrows indicate the direction in which the dot moved. **(B)** Bias and variance of reported orientation. In the bias graphs, positive values indicate a clockwise bias and negative values indicate a counterclockwise bias. The error bars represent 95% confidence intervals (CIs). See Table 1 for the results of the main analysis for all data for the hand and the arm. Notes, lines, and asterisks added to the data in each panel all represent the results of the post-hoc analysis for one of the body part sites: hand or arm. Black lines above the data are significance bars of multiple comparisons where the factor of the stimulus angle at the arm (or at the hand) was analyzed as a dependent mean. Asterisks on the graph represent simple effects for interaction between stimulus dynamics and stimulus angle: horizontally-aligned black asterisks represent significant simple effects of stimulus dynamics at each stimulus angle, while vertically-aligned coloured ones represent those of the stimulus angle for each stimulus dynamics, each with alpha level 0.05.

The reported orientation of the stimuli for most conditions were correct when the stimuli were applied to the hand. The colored radar charts (reported shape) have well-defined peaks along the black dashed line (presented shape) (Fig. 2A). On the other hand, when the stimuli were applied to the arm, performance was degraded, especially with right-tilted stimuli presented at around 135 °.

We calculated bias that represents the mean discrepancy between the reported and veridical angles of the stimuli and variance that represents the variability of responses for each stimulus (Fig. 2B). The bias of each participant (shown in the left two panels of Fig. 2B) was input to repeated measures of the analysis of variance (ANOVA) with three per-participant variables: location (hand, arm), stimulus dynamics (4 types), and stimulus angle (8 values). Multiple comparisons were corrected with Shaffer’s modified sequentially rejective Bonferroni procedure^37^. All main effects and interactions were significant except the stimulus dynamics (see Table 1 for ANOVA details). For the sake of readability, we refrain from considering all these effects in detail. Instead, since the main effects and interactions of location are significant, we report the partial analyses that served to clarify the results for the arm (the bottom-left panel of Fig. 2B) and left out those for the hand (for other results, please check the symbols in the figure along with the legends). In post-hoc analysis, multiple comparisons for the stimulus angle applied to the arm showed significant clockwise bias when the stimulus angle was 157.5° compared to when the angle was 0°–90°, when the stimulus angle was 135° compared to when the angle was 22.5°–90°, and when the stimulus angle was 0 °s compared to when the angle was 22.5°–45° (see the significance bars in the bottom-left panel of Fig. 2B). Note that the results observed for the arm were different from those observed for the hand, both in terms of absolute values and in terms of the pattern of changes due to the stimulus angle. Multiple comparisons for stimulus dynamics applied to the arm did not show significant difference between any pair. In addition, simple effects for interaction between stimulus dynamics and stimulus angle were significant for all stimulus dynamics (noted as red, green, blue, and purple asterisks in the bottom-left panel of Fig. 2B).

**Table 1:** Results from the ANOVAs of Experiment 1.

Effect	df1, df2	For bias data			For variance data		
		F-value	p-value	eta^2^	F-value	p-value	eta^2^
Location (L)	1, 13	33	** < 0.001**	0.03	95	** < 0.001**	0.18
Dynamics (D)	3, 39	1.1	0.4	0.002	21	** < 0.001**	0.05
Angle (A)	7, 91	8.9	** < 0.001**	0.13	4.8	** < 0.001**	0.05
L × D	3, 39	6.9	** < 0.001**	0.01	17	** < 0.001**	0.02
L × A	7, 91	13	** < 0.001**	0.1	6.9	** < 0.001**	0.05
D × A	21, 273	3.5	** < 0.001**	0.04	2.0	** < 0.01**	0.02
L × D × A	21, 273	1.2	0.2	0.01	1.4	0.1	0.01

The same analysis was performed for the variance data (the right two panels of Fig. 2B), and all main effects and interactions were significant. Again, since the main effects and interactions of location are significant, we report the partial analyses of the results for the arm (the bottom-right panel of Fig. 2B). Multiple comparisons for the stimulus angle applied to the arm showed that variance was bigger when the stimulus angle was 135 °s compared to when the angle was 22.5°–67.5°. Multiple comparisons for stimulus dynamics applied to the arm showed that variance was smaller under the static condition (purple line in the graph) than under other conditions (noted as “M+ , M−, Sh > St” in the bottom-right panel of Fig. 2B). In addition, simple effects for interaction between stimulus dynamics and stimulus angle were significant only for dynamic stimuli and not for static stimuli (noted as red, green, and blue asterisks in the bottom-right panel of Fig. 2B).

A major finding is that the accuracy of orientation perception of simple line stimuli differed based on where the stimuli were applied (e.g., to the arm or the hand). Also, the intensity of bias of the perceived angle on the arm was different based on the stimulus angle. These analyses suggest anisotropic distortion of the perceived orientation on the arm: The perceived stimulus angle on the arm was biased inward (i.e., clockwise for the left arm) when the stimulus angle was around 135°; however, it was not biased outward or inward when the stimulus angle was around 45°.

### Experiment 2

The purpose of Experiment 2 was to verify whether the differences in orientation perception between the hand and the arm observed in Experiment 1 could be replicated on the opposite side of the body (i.e., right hand and right arm). Also, we tried to see whether the anisotropic distortion of the perceived orientation on the arm would show a parallel shift from the left arm to the right arm or show a body-centered symmetrical shift.

**Figure 3:**
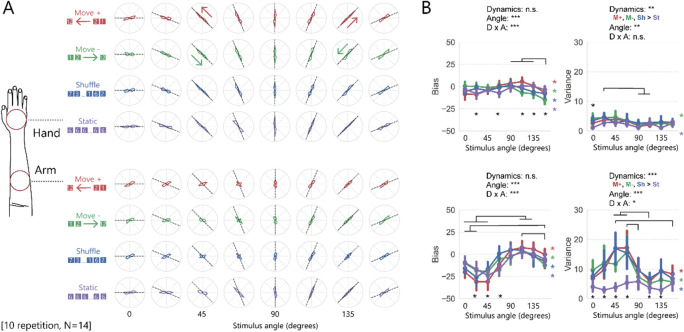
Results of Experiment 2. **(A)** The average rate of the stimulus orientation reported by the participants. **(B)** The bias and variance of the reported orientation. The error bars represent 95% CIs.

Similar to what was observed in Experiment 1, the reported angle of the stimuli was accurate when the stimuli were applied to the hand but not when the stimuli were applied to the arm (Fig. 3). Moreover, the reported angle on the arm showed “body-centrical symmetry”: the perceived orientation on the right arm was biased inward when the stimulus was left-tilted, but it was not biased when the stimulus was right-tilted.

A three-way ANOVA (location, stimulus dynamics, and stimulus angle) showed similar results to Experiment 1, although not in complete agreement (see Table 2 for ANOVA details). The bias and variance of the orientation perception differed based on where the stimuli were applied and the stimulus angle.

**Table 2:** Results from the ANOVAs of Experiment 2.

Effect	df1, df2	For bias data			For variance data		
		F-value	p-value	eta^2^	F-value	p-value	eta^2^
Location (L)	1, 13	37	** < 0.001**	0.03	88	** < 0.001**	0.2
Dynamics (D)	3, 39	0.14	0.9	0.0004	41	** < 0.001**	0.05
Angle (A)	7, 91	12	** < 0.001**	0.14	6.8	** < 0.001**	0.06
L × D	3, 39	1.4	0.3	0.002	24	** < 0.001**	0.03
L × A	7, 91	11	** < 0.001**	0.09	5.3	** < 0.001**	0.03
D × A	21, 273	4.5	** < 0.001**	0.05	2.6	** < 0.001**	0.03
L × D × A	21, 273	1.7	**0.03**	0.02	2.3	** < 0.01**	0.02

In post-hoc analysis with bias data for the arm (see the bottom-left panel in Fig. 3B), multiple comparisons for the stimulus angle showed significant clockwise bias when the stimulus angle was 90°–135° compared to when the angle was 0°–45°, when the stimulus angle was 157.5° compared to when the angle was 0°–22.5°, and when the stimulus angle was 112.5° compared to when the angle was 157.5°. Note that although the analysis results for Experiments 1 and 2 look similar when represented as significance bars, the right-hand condition (Experiment 2) showed a counterclockwise bias for the left-tilted shape, while the left-hand condition (Experiment 1) showed a clockwise bias for the right-tilted shape, as shown in Figs. 2B and 3B. In addition, simple effects for interaction between stimulus dynamics and stimulus angle were significant for all stimulus dynamics.

In post-hoc analysis with variance data for the arm (bottom-right panel in Fig. 3B), multiple comparisons for the stimulus angle showed that variance was bigger when the stimulus angle was 45° or 67.5° compared to when the angle was 90°, 112.5°, or 157.5°; and multiple comparisons for stimulus dynamics showed that the variance was smaller under the static condition than under other conditions. Simple effects for interaction between stimulus dynamics and stimulus angle were significant only for dynamic stimuli and not for static stimuli.

These analyses are in good agreement with the analysis of Experiment 1, suggesting the anisotropic distortion of the perceived orientation occurs both on the left and the right arms, in a body-centrical, symmetrical manner.

### Experiment 3

The purpose of Experiment 3 was to see if and how the observed anisotropic distortion of the perceived orientation on the arm shifts with posture changes. This experiment was inspired by one important study of orientation perception with hands^22^, which reported the huge impact arm posture had on perception of haptic parallelism. Note that our participants’ task was to report the angle of the perceived stimuli at coordinates fixed in space (i.e., the surface of the stimulator).

**Figure 4:**
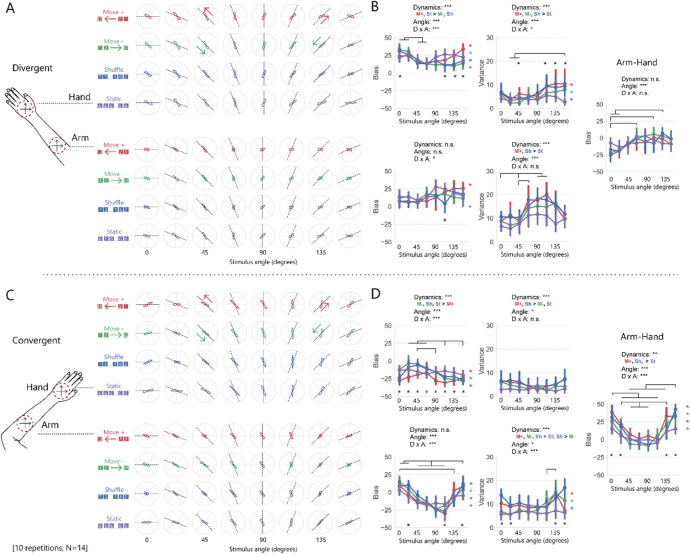
Results of Experiment 3. **(A)** The average rate of the stimulus orientation reported by the participants under divergent posture. The schematic illustration of axes drawn on the hand or arm shows the coordinates (fixed in space) that the participants were asked to use to report perceived shape. **(B)** The bias and variance of the reported orientation under divergent posture. The rightmost panel shows the angle bias reported on the arm minus the angle bias reported on the hand. The error bars represent 95% CIs. **(C)** The average rate of the stimulus orientation reported by the participants under convergent posture. **(D)** The bias and variance of the reported orientation under convergent posture. The rightmost panel shows the angle bias reported on the arm minus the angle bias reported on the hand.

Here, in contrast to the previous two experiments in which the participants’ hands were oriented straight ahead, the reported angle of the stimuli was biased even when the stimuli were applied to the hand (Fig. 4A,C). There were variations depending on the dynamics and angle of the stimulus but, basically, the reported angle was biased clockwise in the divergent posture (positive bias as shown in the top-left panel of Fig. 4B) and counterclockwise in the convergent posture (negative bias as shown in the top-left panel of Fig. 4D).

A four-way ANOVA (posture, location, stimulus dynamics, stimulus angle) for bias data showed significant main effects of posture, stimulus dynamics, and stimulus angle (not location), while that for variance data showed significant main effects of location, stimulus dynamics, and stimulus angle (not posture) (see Table 3 for ANOVA details). Importantly, the interaction between posture and the stimulus angle and that between posture, location, and the stimulus angle were significant for both bias and variance data, supporting the possibility that the observed anisotropic distortion of the perceived orientation on the arm shifted with posture changes.

**Table 3:** Results from the ANOVAs of Experiment 3.

Effect	df1, df2	For bias data			For variance data		
		F-value	p-value	eta^2^	F-value	p-value	eta^2^
Posture (P)	1, 13	90	** < 0.001**	0.4	3.7	0.08	0.02
Location (L)	1, 13	2.0	0.2	0.003	88	** < 0.001**	0.13
Dynamics (D)	3, 39	3.3	** < 0.05**	0.002	25	** < 0.001**	0.04
Angle (A)	7, 91	6.1	** < 0.001**	0.02	4.9	** < 0.001**	0.02
P × L	1, 13	40	** < 0.001**	0.03	2.2	0.2	0.004
P × D	3, 39	12	** < 0.001**	0.005	1.4	0.3	0.001
P × A	7, 91	6.0	** < 0.001**	0.02	7.4	** < 0.001**	0.02
L × D	3, 39	2.1	0.1	0.001	2.3	0.1	0.003
L × A	7, 91	5.9	** < 0.001**	0.01	3.8	** < 0.01**	0.01
D × A	21, 273	7.4	** < 0.001**	0.02	2.5	** < 0.001**	0.009
P × L × D	3, 39	4.0	** < 0.03**	0.002	0.14	0.9	0.0001
P × L × A	7, 91	29	** < 0.001**	0.03	4.7	** < 0.001**	0.01
P × D × A	21, 273	1.2	0.3	0.004	1.4	0.1	0.006
L × D × A	21, 273	2.0	** < 0.01**	0.005	2.1	** < 0.01**	0.009
P × L × D × A	21, 273	1.5	0.07	0.004	1.3	0.2	0.005

Since the performance of orientation perception in the baseline hand condition also changed with the change in posture, we decided to verify the performance of orientation perception of stimuli applied to the arm after removing the effect of this baseline. For each participant, response bias for each condition (i.e., each combination of posture, stimulus dynamics, and stimulus angle) applied to the hand was subtracted from that for each condition applied to the arm (the rightmost panels in Fig. 4B,D), and entered into a three-way ANOVA (posture, stimulus dynamics, stimulus angle). The analysis for subtracted bias data showed significant main effects of posture and the stimulus angle (not stimulus dynamics). Importantly, interaction between posture and the stimulus angle were again significant (see Table 4 for ANOVA details). That is, the possibility remained, after subtraction of the baseline bias observed with the hand, that the observed anisotropic distortion of the perceived orientation “on the arm” shifted with posture changes. In addition, interactions between stimulus dynamics and stimulus posture and angle were significant.

**Table 4:** Results from the ANOVAs for the difference data of Experiment 3.

Effect	df1, df2	For bias data		
		F-value	p-value	eta^2^
Posture (P)	1, 13	40	** < 0.001**	0.12
Dynamics (D)	3, 39	2.1	0.1	0.004
Angle (A)	7, 91	5.9	** < 0.001**	0.06
P × D	3, 39	4.0	** < 0.03**	0.01
P × A	7, 91	29	** < 0.001**	0.14
D × A	21, 273	2.0	** < 0.001**	0.02
P × D × A	21, 273	1.5	0.07	0.02

In post-hoc analysis of data for each posture, multiple comparisons of the stimulus angle under divergent posture (the rightmost panel in Fig. 4B) showed significant clockwise bias when the angle was 67.5°, 112.5°, or 135° compared to when the angle was 0°, and when the angle was 135° compared to when the angle was 22.5°. Stimulus angle under convergent posture (the rightmost panel in Fig. 4D) showed significant clockwise bias when the angle was 135° compared to when the angle was 45°–90°, when the angle was 157.5° compared to when the angle was 45°–135°, when the angle was 0° compared to when the angle was 22.5°–112.5°, and when the angle was 22.5° compared to when the angle was 45°–90°. As can be seen from the difference in the significance bars between the rightmost panel of Fig. 4B and that of Fig. 4D, the distribution of subtracted bias data across stimulus angle depended on the posture.

### Experiment 4

The purpose of Experiment 4 was to see if and how the observed anisotropic distortion of the perceived orientation on the arm occurred at different skin sites on the arm. In this experiment, participants were asked to put their arm on the stimulator in palm-side and palm-up postures in addition to the palm-down posture. We could not test the hand condition in these postures because it was difficult to ensure contact between the surface of the stimulator and the dorsal and lateral sides of the hand.

**Figure 5:**
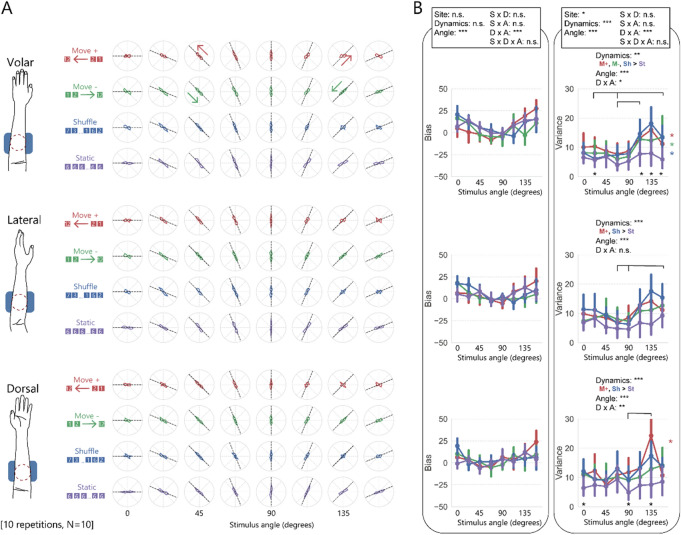
Results of Experiment 4. **(A)** The average rate of the stimulus orientation reported by the participants under volar, lateral, and dorsal conditions. **(B)** Biases and variances of the reported orientations. The error bars represent 95% CIs. Notes in the box above panels represent the results of the main analysis for all stimulus sites, while notes above each panel represent the results of the post-hoc analysis for only one of the stimulus sites. A partial analysis for each site was not conducted for the bias data.

Results are shown in Fig. 5. A three-way ANOVA (stimulus site, stimulus dynamics, stimulus angle) for bias data showed the significant main effects of the stimulus angle but not that of the stimulus site or stimulus dynamics. Any interaction with the stimulus site was also not significant, suggesting a minor effect of skin-site difference on the bias of shape perception on the arm (see Table 5 for ANOVA details). Since significant differences related to skin-site were not reported, we did not conduct a partial analysis for each site as we did in Experiments 1 through 3. Multiple comparisons of the stimulus angle for all bias data, including results for all sites, showed clockwise bias when the angle was 0° or 157.5° compared to when the angle was 90°, and when the angle was 0° or 22.5° compared to when the angle was 67.5°. This analysis result is not intuitive in the sense that observed change in the bias was not identical to that observed in Experiment 1. It is possible that the overall trend was not observed above the detection threshold of the analysis due to the large amount of variability between individuals and/or between different stimulus site conditions. This may reflect the difficulty of performing the experiment in awkward postures.

**Table 5:** Results from the ANOVAs of Experiment 4.

Effect	df1, df2	For bias data			For variance data		
		F-value	p-value	eta^2^	F-value	p-value	eta^2^
Site (S)	2, 18	3.4	0.06	0.004	4.2	** < 0.05**	0.009
Dynamics (D)	3, 27	0.90	0.5	0.004	11	** < 0.001**	0.06
Angle (A)	7, 63	5.3	** < 0.001**	0.1	7.3	** < 0.001**	0.08
S × D	6, 54	0.85	0.5	0.004	0.82	0.6	0.002
S × A	14, 126	1.7	0.06	0.02	1.6	0.08	0.01
D × A	21, 189	2.9	** < 0.001**	0.07	2.5	** < 0.001**	0.03
S × D × A	42, 378	1.2	0.2	0.02	1.0	0.5	0.02

A three-way ANOVA for variance data showed the significant main effects of the stimulus site, stimulus dynamics, and stimulus angle, however, any interaction with the stimulus site was not significant. We additionally conducted multiple comparisons of the stimulus angle for all variance data (those for each site is shown in Fig. 5B). A bigger variance was observed when the angle was 112.5° or 135° compared to when the angle was 90 °s. In addition, simple effects for interaction between stimulus dynamics and stimulus angle were significant only for dynamic stimuli and not for static stimuli.

## Discussion

Experiments 1 and 2 showed an unexpected illusion of perceptual distortion of orientation for a certain angle of stimulus: An inwardly-inclined line shape was perceived as excessively inwardly-inclined. This illusory perception occurred on the arms but not on the palms. When the stimulus was presented dynamically, the perceived orientation was more unstable than when the stimulus was presented statically, but the response bias (intensity of illusion) was not significantly different. Experiment 3 showed that changing the posture of the arm changed the angle at which this illusion occurred. Experiment 4 showed a minor effect of varying the skin site on the observed anisotropic bias in the perceived orientation of the stimuli applied to the arm.

There are both similarities and differences between the present findings and previous studies that have examined orientation sensitivity, especially studies that investigated haptic parallelism (e.g.,^22–30^). First of all, there are significant differences in experimental procedures. In those previous studies, participants touched bars (e.g., aluminum rods) with their hands and were asked to position a test bar parallel to a reference bar. In other words, their task required bodily movement (active perception), and had nothing to do with perception on the arm itself.

Secondly, current findings are not fully explained by the findings of previous studies. One famous finding in previous studies, a haptic oblique effect, is that deviations of the reported/matched angle for the oblique reference bar were substantially bigger than that for the vertical and horizontal reference bars^23,25–28,31^. Indeed, we observed a huge change depending on stimulus angle (Figs. 2B, 3B bottom panels), but the way change occurs seems to be different from the typical oblique effect: poor performance on certain angles (right oblique error for the left arm, left oblique error for the right arm) was observed instead of roughly equal right and left oblique errors. Our tentative understanding here is that the observed anisotropic distortion in the perceived orientation of the stimuli applied to the arm is a new illusion in the sense that it cannot be fully explained by the haptic oblique effect.

Another major finding of previous studies might be more closely related to the results of the present study. Stimuli that are perceived as haptically parallel can deviate significantly from stimuli that are physically parallel (e.g.,^22,25,27,29,30^), and this is related to angle shifts (bias). Reported deviations from parallelity are significant—as large as 90° in some cases^25^—which reminds us of the effect size of our illusion. Figure 2 in^27^ shows that a counterclockwise bias was observed when participants were asked to rotate the bars in front of their body to be parallel to the fixed bars placed on the right side of their body. In addition, this bias appeared to be greater for inwardly-tilted stimuli (the angle at which we observed the illusion) than for outwardly-tilted, vertical, or horizontal stimuli. This suggests the possibility that the inwardly-tilted shape on the right side of the body is equivalent to a more inwardly-tilted shape when represented in front of the body. This is consistent with our observation of a counterclockwise bias for the left-tilt shape in Experiment 2 in which participants touched the stimuli with their right arm placed at the right side of their body center. If the phenomena reported in the previous studies and those in the current study occurred based on or sharing the same mechanism, we can speculate that this tactile spatial distortion occurs in a general spatial representation of touch that is not restricted to the specific skin site and is used both for sensory perception and motor action. This is an issue awaiting further investigation.

Our Experiment 3 found the effect of arm posture on orientation perception. This is consistent with the previous study about haptic parallelism with hands^22^, which was the source of the idea for our experimental conditions. In their work, Kappers and Viergever tested to find out what is haptically parallel under different postures, and they observed the strong effect a hand-centered egocentric frame of reference has on a perceived angle (i.e., bias). We also observed that a perceived angle on the hand was strongly influenced by a hand-centered somatotopic frame even though the participants were explicitly asked to answer about the perceived angle in terms of the spatiotopic frame fixed in space (i.e., the surface of the stimulator) (Fig. 4B,D top-left panels). The bias was consistent with the angle shift of the somatotopic frame from the spatiotopic frame due to the change in posture (+ 45° for the divergent condition, − 45° for the convergent condition) in terms of direction, although the shift values did not match perfectly. Meanwhile, the pattern of the perceived angle bias on the arm was complex but seemed to reflect the angle bias observed on the hand under both divergent and convergent postures and the illusion observed on the arm under normal posture (Fig. 4B,D bottom-left panels). Therefore, we subtracted the hand bias from the arm bias to see how the illusion changed according to posture (Fig. 4B,D rightmost panels). If the reference frame of the illusion is spatiotopic, the singularity angle (angle at which the bias takes an extreme value) should be independent of the posture condition. If, on the other hand, the reference frame is somatotopic, the singularity should shift with the posture change. The bias results of Experiments 1 and 3 suggest that changing the posture changed the angle at which this illusion occurred. Our tentative understanding here is that the reference frame of the observed illusion on the arm is not purely a body-centered spatiotopic frame; rather, it is a frame intermediate to a spatiotopic and hand-centered somatotopic one. Though our findings are not derived from the conclusions of previous studies on haptic parallelism, they are not inconsistent with them^22,26^ either.

We presented the stimuli using the stimulator rather than manually to manipulate stimulus dynamics: dots appear one by one in one direction, in random order, or all at once. As a result, the intensity of illusion, the value of bias, did not differ significantly depending on the dynamics of the stimuli. Multiple comparisons for stimulus dynamics applied to the arm showed that there were no significant differences between any dynamics pairs. Since simple effects of stimulus dynamics were significant only at stimulus angle where discrepancy between the reported and veridical angles of the stimuli was big (represented as horizontally-aligned black asterisks in Figs. 2B, 3B), there remains a possibility that stimulus dynamics may influence bias data but just not visible in current results due to a floor effect. Still, there is no strong evidence at this moment that prediction or pooling affected this illusion. Some previous studies about distance perception reported a different trend regarding the difference between static stimuli and dynamic stimuli: distance perception was much more imprecise (i.e., large bias) for simultaneous stimulation than for sequential stimulation^9,14,15^. This apparent discrepancy might actually be due to the differences in focused features (angle or distance) and/or stimulus presentation between previous studies and ours. In previous studies, the same distance was presented and compared between simultaneous stimuli and sequential stimuli. In our dynamic (i.e., sequential) conditions, the distance between successively presented dots was relatively small within a short time window compared to that with the static (i.e., simultaneous) condition, so it was not possible for participants to get a complete picture of the shape of the presented stimuli all at once. This might distort the represented angle of the stimuli in dynamic conditions and result in a performance equal to that of the static condition. Regarding the differences among dynamic stimuli, one previous study reported that when the line stimuli were applied to the arm by seven tactors, judgments of perceived length, smoothness, straightness, and even spatial distribution of the line stimuli were very similar to that of a different presentation pattern of motion^16^. This is consistent with our observation of minor differences between the results of bias with three types of dynamic stimuli.

Several factors may contribute to the observed illusion. One plausible explanation that has been suggested in previous studies is the effect of the distribution and density of mechanoreceptors. There have been a number of reports on discrepancies between tactile space and physical space^1–3,6–11,14,15^. For example, touch distances along the arm were perceived to be longer than those across the arm^3,7^, and touch locations on the arm were perceived to be closer to the wrist and the elbow^2,10^. Some studies investigated localization for touch and even pain on the dorsal surface of the hand and arm, and found that stimulus localization was more accurate (smaller uncertainty intervals) in the short axis than in the long axis^10,11^. The current study differs from those studies in that we are reporting a phenomenon wherein localized distortions occur rather than uniform stretching/shifting of perceived spaces. Nevertheless, the explanation proposed in the previous studies^2,5,10,11^ in terms of the distribution and density of mechanoreceptors^12,13^ may also partially explain our results. As problematized in one of the previous studies, “the skin is far from a uniform receptive organ”^2^. We carefully chose the central area of the forearm to stimulate; this area is not close to the wrist or the elbow, but the distribution of receptors may still differ among the stimulated skin areas. For example, we can even hypothesize that there are fewer receptors on the thumb side than on the little finger side on the skin near the wrist, and there are fewer receptors on the little finger side than on the thumb side on the skin near the elbow. Indeed, one study reported sensitivity difference due to the skin site on the arm: intensity discrimination was better when the vibrotactile stimuli were applied to the skin of the arm on the thumb side than when the stimuli were applied to the skin of the arm on the little finger side^18^. Another study reported varying localization acuity at different distances along the arm^10^. Since we observed that the illusion shifted based on the somatotopic (i.e., skin) reference frame, this hypothesis is not unlikely. Future detailed neurophysiological research about mechanoreceptor distribution across the arm may provide valuable insight into this illusion. Still, the reason the perceived angle is biased in a certain direction of rotation (clockwise with the left arm, counterclockwise with the right arm) remains an open question.

Another possibility relates to a potentially incomplete contact condition. Since we observed body-centrical symmetry in a perceived shape pattern between the left arm (Exp. 1) and the right arm (Exp. 2), the observed illusion is very unlikely to reflect the characteristics of the braille stimulator itself (e.g., it is unlikely that the pins located closer to the thumb rose to the “on” position with less force than the pins located closer to the little finger). However, there remains sufficient possibility that the state of contact between the arm and the stimulator was not physically uniform since arms are not perfectly cylindrical in shape, nor is their fleshy tissue uniformly distributed. Thus, it is possible that the state of contact with the flat braille display was not the same between the wrist side and the elbow side (or the thumb side and the little finger side). We tested the stability of the illusion by changing the skin location in Experiment 4 in order to address these possible problems (including the different distribution of receptors and contact states). Unfortunately, the obtained results did not show a clear illusion: multiple comparisons of the stimulus angle showed a similar but not identical pattern of significant differences as in Experiment 1. This might reflect the difficulty of the task, since the participants in this experiment were only tested for perception of stimuli on the arm, and the postures were a bit difficult to adopt. The effect of difference in attentional state might be relevant here, and this will be discussed later. Nevertheless, we did not find any positive evidence showing that the illusion had been significantly affected by changing stimulated skin locations. In other words, the bias in orientation perception of simple line stimuli did not change even when the distribution of receptors and contact conditions were presumably changed by switching the skin site where the stimuli were applied. Further investigation is warranted to develop a device that can stimulate heterogeneous points on the skin of the arm uniformly.

An alternative, although not exclusive, explanation of observed illusion could be an attentional effect. One previous study found effects of top-down and bottom-up attention on stimulus localization acuity on the arm^32^. Participants could localize the input stimuli more accurately when they were explicitly instructed to direct their attention to the stimulated area, compared to when they directed their attention to the entire arm. Also, they could localize more accurately when stimulation of a certain area was more probable than that of another area. Based on these findings, one may hypothesize that it is generally difficult for the brain to allocate attention to the angle at which our illusion occurs. Our results, however, do not necessarily support this hypothesis. We manipulated the dynamics of the stimuli, which should have affected the attentional state, but it made only minor differences to the intensity of the observed illusion. This hypothesis still awaits further empirical validation.

When we look at the pattern of response variances between different stimulus dynamics, static stimuli caused a smaller variation in the perceived angle compared to dynamic stimuli, and this trend was robustly observed throughout Experiments 1 to 4. This suggests that participants were able to perceive the orientation stably when the stimulus extended over a larger area even if it was presented for a short period of time, 200 ms. As described above, the distance between successively-presented dots was relatively small in our dynamic conditions. Although the end-to-end distance of all the stimulus dots was set to be longer than the two-point discrimination threshold for the forearm^19^, the dots were presented under this threshold within a short time window, and this might have caused uncertain perception in dynamic conditions. Notably, we did not observe a better performance with the shuffle condition than with the move conditions even though the distance between the successively-presented dots was stochastically longer in the shuffle condition. It seems that the brain can estimate the shape of the input on the arm by integrating the spatiotemporal input over a certain amount of time. To summarize, the effect of the size of the stimulus was evident in the accuracy of orientation estimation on the arm, while that of the trackability of the stimulus remains obscure. It can be speculated that the receptive field size of the orientation filter for shape estimation is larger when processing stimuli applied to the arm than when processing stimuli applied to the hand, which seems reasonable since there is also a difference in receptive field size for signal detection between the hand and the arm.

Interestingly, not only the pattern of the bias but also that of the variance differed depending on the angle of the stimulus applied to the arm. The results of Experiments 1 and 4 showed a variance peak at around 135°, while those of Experiment 2 showed a peak at around 45°. That is, there is a common ambiguity in perception of inwardly-inclined shapes in body-centered coordinates when the arm is oriented straight ahead, and this trend was observed regardless of the skin site (volar, lateral, and dorsal sides of the arm). Analysis of simple effects for interaction between stimulus dynamics and stimulus angle suggests that this anisotropic distortion of the stability of orientation perception is observed only with dynamic stimuli. Since this anisotropic distortion of accuracy was not observed with static stimuli, it is unlikely to be a simple consequence of poor contact conditions with inwardly-inclined stimuli. Though this hypothesis remains highly speculative at the moment, there might be differences between the hand and the arm in the temporal integration window and/or in the computation of integrating spatio-temporal information. Further research is warranted with an ideal device that can present stimuli at higher refresh rates to enable the presentation of analogous stimuli while maintaining the characteristics of the different dynamics. Both anisotropic distortion in the perceived angle of the shape and anisotropically-distorted stability of shape perception await further investigation.

## Methods

### Participants

Thirty naïve participants (eight men; aged 20 to 48 years; mean age 30.6 years; all right-handed) with normal tactile sensitivity (self-reported) participated in the experiments, with partial overlaps of participants across experiments. All gave written informed consent before the start of the experiment. The NTT Communication Science Laboratory Research Ethics Committee approved the recruitment of the participants and the experimental procedures, which were conducted in accordance with the Declaration of Helsinki.

The main purpose of the experiments was to see the difference in orientation perception when a tilted-line shape was applied to the palm of the hand and when the same shape was applied to the volar surface of the arm. A power analysis (MorePower^33^) showed that the number of participants needed to achieve 80% power for the main effect of the stimulus angle (8 levels in factor) and a within-participants interaction (for the factors of the stimulus site (hand vs. arm) and stimulus angles (2 and 8 levels)) using a repeated measures ANOVA with eta^2^ = 0.14^34^, power of 1–β = 0.8, and a two-tailed α = 0.05 is 14 per group. We recruited this number of participants for Experiments 1, 2, and 3. The last additional experiment was conducted to see the effect of the skin site on the perceived shape on the arm. The same power analysis was done for a 3 × 8 interaction for the factors of the skin site (volar, lateral, and dorsal) and stimulus angles, and the calculated number of participants was ten. We recruited this number of participants for Experiment 4.

### Apparatus

Tactile stimuli were presented to the participants with a piezoelectric braille display (stimulator, hereafter) (Dot View DV-2, KGS, Japan), which was the same as in our previous study^35^. The stimulator has an array of pins with a diameter of 1.3 mm and an inter-pin distance of 2.4 mm. Each pin can be switched independently to either the “on” position (maximum 0.7-mm normal displacement or less when damped by the contacting hand) or the “off” position (no displacement), and the status of the pins (“on” or “off”) was updated every 100 ms. Note that “on”-pins did not vibrate (which is typical of other braille systems such as OPTACON (Telesensory Systems, Palo Alto, CA)) but remained stationary during the specified period with a rise time of 15 ms.

The stimuli were 12 aligned dots presented within a circular area 32 pins in diameter (74.4 mm), and their angle was one of eight possibilities (Fig. 1C). This size of the stimuli is bigger than the two-point discrimination threshold for the forearm^21^. The dots were presented sequentially in one direction [“move + condition”], in the opposite direction [“move-”], in random order [“shuffle”], or presented all at once [“static”] within 2 s. Partially spatially-overlapping with adjacent dots, each dot consisted of four to six “on” pins and appeared for 0.1 to 0.2 s. These variations were unavoidable due to the innate characteristics of the stimulator.

In Experiments 1–3 (Fig. 1A), each participant sat at a table and touched the stimulator with the volar surface of their hand or forearm. In Experiment 4, they touched the stimulator with the volar, lateral, and dorsal surface of their forearm. The stimulator was located to the right/left of the body midline when the participant used their right/left hand/arm so that they could comfortably place their palm/arm. The stimulator maintained contact with the participants’ skin throughout the experiment. Participants responded by pressing a keyboard with their free hands. They performed the tasks with their eyes open to maintain their arousal level, facing forward where a response map (Fig. 1B) was presented. The display parts of the stimulators were occluded from the participants’ view by a blackboard.

### Experimental design

The experimental setup and procedure were nearly identical between experiments and only differed in where and how the participants touched the stimulator. At the beginning of each block, the braille stimulator presented a cross shape in the center of the display area to remind participants of the coordinate center of the stimuli. The participants were asked to press a key when they were ready. After the key press, all the pins went into the “off” position and a beep sounded to signal the start of the trial. The dot stimuli started after a 500-ms blank period. After presenting stimuli for 2000 ms, all the pins went into the “off” position. The participants were asked to report the perceived shape of the stimuli with respect to the stimulator surface (coordinate fixed in space, not fixed to their hands/arms) by pressing two keys based on the response map (e.g., the participant pressed “4” and “R” when they perceived a vertical angle). Whether the line shape was perceived symmetrically remained obscure, so both ends of the perceived shape (rather than one angle) were recorded. The participants knew that the presented stimuli passed the center of the stimulus area, but they did not know that the stimuli would always be in (approximately) straight lines. No feedback signal was provided. After their response, the beep sounded to signal the start of the next trial. Each block lasted around five minutes and contained 32 trials of different combinations of eight angle conditions {0°, 22.5°, 45°, 67.5°, 90°, 112.5°, 135°, 155.5° in which stimuli at 0° were defined with respect to the front edge of the stimulator placed parallel to the table} and four stimulus presentation modes {move + , move −, shuffle, static}. The presentation order was randomized within each block.

In Experiment 1, 14 participants participated for the left-hand and arm conditions with their hands/arms oriented straight ahead (normal posture). In Experiment 2, another group of 14 participants participated for the right-hand and arm conditions with normal posture. In Experiment 3, another group of 14 participants participated for the left-hand and arm conditions with their hands/arms rotated outward (divergent) and rotated inward (convergent posture). In these experiments, the participants touched the stimulator with the volar surface of their hand or forearm. In Experiment 4, ten participants participated for the left arm conditions in which volar (palm-down posture), lateral (palm-side), and dorsal (palm-up) skin sites touched the stimulator.

Before the experiment, the participants conducted a practice session in which they familiarized themselves with the stimuli and the task by looking at and touching the stimuli and by pressing keys to report perceived shapes. All the participants finished eight to ten blocks not including practice blocks; i.e., each condition (a combination of touched location × stimulus angle condition × stimulus presentation mode) was tested eight to ten times. A block was excluded from later analysis when participants reported that they had failed in signal detection (not discrimination) (e.g., due to poor hand placement). The total experiment time including practice and breaks for each participant was around 3 h for Experiment 1, 3 h for Experiment 2, 5 h for Experiment 3, and 4 h for Experiment 4.

### Data analysis

In analysis, distributions of the perceived orientation of the stimuli were calculated for each condition for each participant: the deviation between the actual shape and the reported shape (bias) and the variance of the reported angle were calculated (variance). Graphs show the averaged data across participants, with 95% CIs calculated using a bootstrapping method^36^. A repeated measures ANOVA was conducted for the calculated bias and variance of each participant in each condition. The p-values of interaction and the main effects in these analyses were corrected based on Shaffer's Modified sequentially rejective Bonferroni procedure^37^.
